# PorthoMCL: Parallel orthology prediction using MCL for the realm of massive genome availability

**DOI:** 10.1186/s41044-016-0019-8

**Published:** 2017-01-10

**Authors:** Ehsan Tabari, Zhengchang Su

**Affiliations:** Department of Bioinformatics and Genomics, The University of North Carolina at Charlotte, 9201 University City Blvd, Charlotte, NC 28223, USA

**Keywords:** Algorithms, Sequence alignment, Orthologous Genes, Software

## Abstract

**Background:**

Finding orthologous genes among multiple sequenced genomes is a primary step in comparative genomics studies. With the number of sequenced genomes increasing exponentially, comparative genomics becomes more powerful than ever for genomic analysis. However, the very large number of genomes in need of analysis makes conventional orthology prediction methods incapable of this task. Thus, an ultrafast tool is urgently needed.

**Results:**

Here, we present PorthoMCL, a fast tool for finding orthologous genes among a very large number of genomes. PorthoMCL can be run on a single machine or in parallel on computer clusters. We have demonstrated PorthoMCL’s capability by identifying orthologs in 2,758 prokaryotic genomes. The results are available for download at: http://ehsun.me/go/porthomcl/.

**Conclusions:**

PorthoMCL is a fast and easy to run tool for identifying orthology among any number of genomes with minimal requirements. PorthoMCL will facilitate comparative genomics analysis with increasing number of available genomes thanks to the rapidly evolving sequencing technologies.

## Background

Orthologs are genes in different species derived from the last common ancestor through speciation events. Orthologous genes generally share the same biological functions in their host genomes. Therefore, identification of orthologous genes among a group of genomes is crucial to almost any comparative genomic analysis [1]. In contrast, paralogs, which are genes that are resulted from gene duplication within a species, may have different functions, though their sequences can be highly conserved. Depending on whether duplication occurred before or after speciation, they are called outparalogs or inparalogs, respectively [2]. Thus, a major challenge in predicting orthologs of a gene is differentiating its orthologs from the orthologs of its paralogs.

Furthermore, due to the rapid advancement in sequencing technologies, sequencing a prokaryotic genome now occurs at an unprecedentedly fast speed and low cost. As a result, tens of thousands of prokaryotic genomes have been fully sequenced, and this number will soon reach hundreds of thousands. The availability of a large number of completed genomes makes comparative genomics an increasingly powerful approach for genome annotations, thereby addressing many important theoretical and application-based problems. However, the rate at which genomes are sequenced outpaces that at which CPU speed increases. This poses a great challenge in comparative genomics that requires faster algorithms or adaptation of existing tools in parallel environments.

OrthoMCL [3] is one of the most widely used algorithms for predicting orthologous genes across multiple genomes. Similar to many other orthology prediction algorithms [4, 5], OrthoMCL is based on reciprocal best hits in all-against-all BLAST searches [6] of complete proteomes of the genomes followed by applying the Markov Clustering algorithm (MCL) [7] to a weighted graph constructed based on these best hits [7, 8]. Specifically, OrthoMCL represents genes as nodes in the graph, and connects two nodes/genes by an edge if there are a pair of reciprocal best hits with a similarity greater than a cutoff. The weight of the edges is a normalized score ($\bar{w}$) based on the E-values of the reciprocal hits. This score for genes *x_A_* and *y_B_* in genomes *A* and *B*, respectively, is calculated using the following formulas:

(1) $$w\left(x_{A},y_{B}\right)=-\frac{\log_{10}\mathrm{Evalue}\left(x_{A}\to y_{B}\right)+\log_{10}\mathrm{Evalue}\left(y_{B}\to x_{A}\right)}{2}$$

(2) $$\bar{w}\left(x_{A},y_{B}\right)=\frac{w\left(x_{A},y_{B}\right)}{{\mathrm{average}}_{\forall \alpha ,\beta }\left(w\left(\alpha_{A},\beta_{B}\right)\right)}$$

Similarly, within-genome reciprocal hits that have a better normalized score than between-genomes hits are identified as paralogs [3]. Ortholog and paralog groups are then identified by finding the heavily connected subgraphs using the MCL [7]. However, OrthoMCL relies on a relational database system to store the BLAST results and issues SQL commands to find reciprocal best hits, making it computationally inefficient when the number of genomes becomes large.

To overcome this problem and to speed up the method further, we developed PorthoMCL, a parallel orthology prediction tool using MCL. In addition to the parallelization, our sparse file structure that is more efficient makes PorthoMCL ultrafast and highly scalable. Furthermore, PorthoMCL is platform independent, thus can be run on a wide range of high performance computing clusters and cloud computing platforms.

## Implementation

### Workflow

**Fig. 1:**
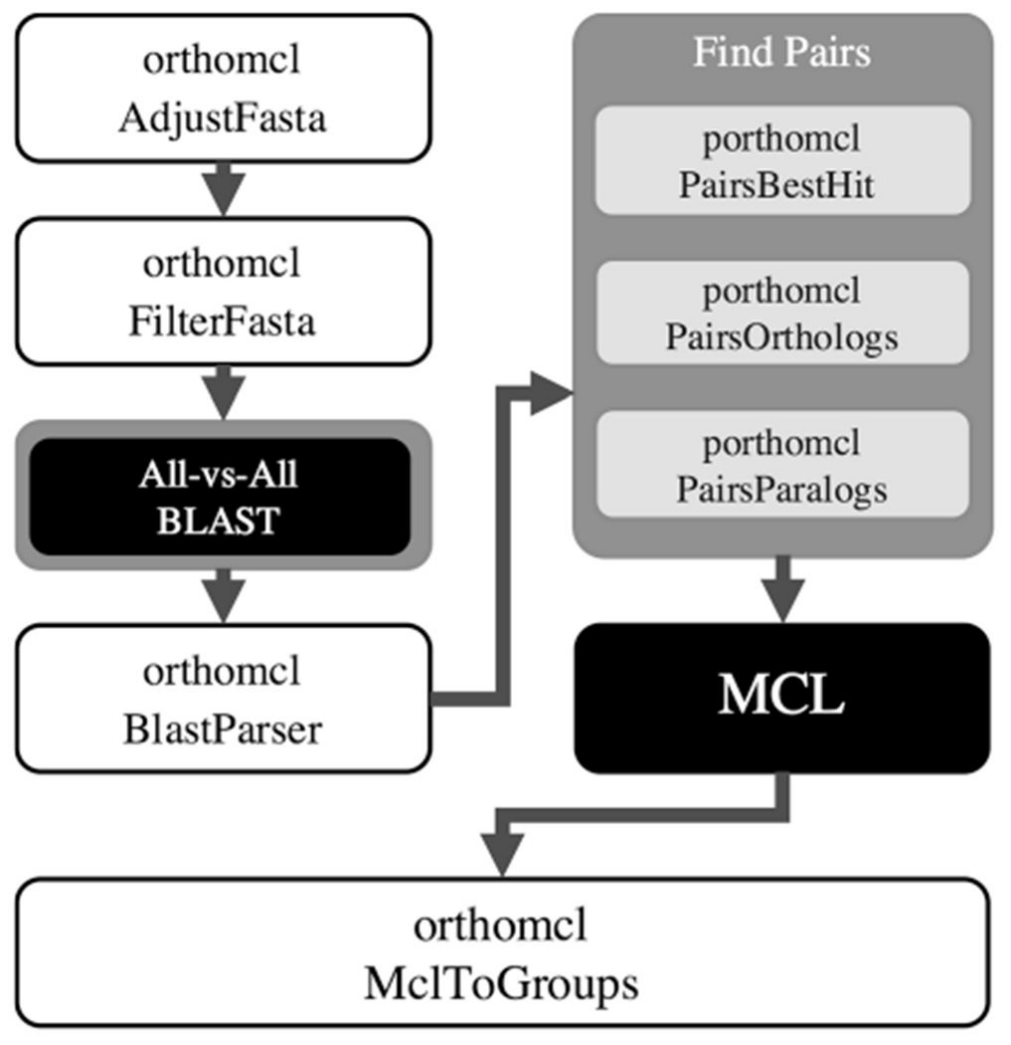
Flowchart of PorthoMCL. Original OrthoMCL steps are shown in white, and PorthoMCL steps are in grey shades. Black boxes are the externalapplications that PorthoMCL requires

The workflow of PorthoMCL is similar to that of OrthoMCL (Fig. 1). However, instead of depending on an external database server, PorthoMCL uses a sparse file structure for more efficient data storage and retrieval. In addition, we parallelized all the computationally intensive steps of OrthoMCL. First, PorthoMCL conducts all-against-all BLAST searches in parallel by performing individual-against-all BLAST searches for every genome independently. Second, it identifies the best between-genomes BLAST hits for each two genomes *A* and *B* in parallel by scanning the individual-against-all BLAST results. The BLAST hit for the gene *x_A_* in genome *B* (*x_A_* → *y_B_*) is considered to be the best hit if the E-value for *x_A_* to gene *y_B_* is the best Evalue for all the searches of *x_A_* for genes in genome *B* with E-value/match-percentage better than the threshold. This step results in a single best hit file for each genome, and a self-hit file for paralogy-finding. Third, the algorithm finds reciprocal best hits between every two genomes and calculates the normalized score in parallel using Formula 2. This is the most computationally intensive step in the algorithm, so we used a sparse file for storage in addition to parallel processing, similar to the strategy used in orthAgogue [9]. Specifically, for each parallel process, PorthoMCL loads at most two best-hit files at the same time to reduce the memory footprint, and every best-hit file is only loaded once to lower the I/O costs. Finally, PorthoMCL finds within-genomes reciprocal best hits and normalizes the score with the average score of all the paralog pairs that have an orthologs in other genomes.

These step are embarrassingly parallel computing problems and do not require shared memory, process coordination or data exchange platform [10] as used in orthAgogue. Hence, these steps are readily designed to be executed in parallel on a variety of high performance computing (HPC) environments. However, these steps are not totally independent as each step needs the output of the preceding step. The output of these steps are eventually collated to construct a sequence similarity graph that is then cut by the MCL program to predict orthologous and paralogous gene groups.

### High performance computing support

PorthoMCL is designed to predict orthologs in a very large number of sequenced genomes in a HPC environments, such as computing clusters or cloud computing platforms without the need of a database server or Message Passing Interface, which is an advantage over OrthoMCL and orthAgogue. We have included a TORQUE script in the repository to facilitate its use in such environments. However, PorthoMCL also runs on a desktop or a server with minimal requirement using the provided wrapper script.

## Results

To compare the computational efficiency of PorthoMCL and OrthoMCL, we applied the two programs to 10, 50, 100 and 500 randomly selected bacterial genomes. As OrthoMCL was not implemented for parallel computing, we ran both programs on a single computing node with four cores and 32GB of RAM to make the comparison fair. As shown in Table 1, PorthoMCL outperformed OrthoMCL in all sizes of datasets in runtime, and it is noteworthy noting that OrthoMCL failed to handle the data size of 500 genomes due to a memory error.

To illustrate the power of PorthoMCL, we applied it to 2,758 sequenced bacterial genomes obtained from GenBank using their annotated protein sequences. These genomes contain a total of 8,661,583 protein sequences with a median length of 270 amino acids. These sequences serve as both the query and the database for all-against-all BLAST searches. For this application, PorthoMCL split the query sequences into smaller files each containing about 10,000 sequences, and ran in the parallel mode on a cluster with 60 computing nodes (each node has 12 cores and 36GBs of RAM). PorthoMCL finished the job in 18 days, of which it spent 11 and 7 days on BLAST searches and the remaining steps that would have taken 549 and 1,634 days, respectively, if run on a single node. In contrast, OrthoMCL could not finish the job after 35 days running on a database server with 40 cores and 1TBs of RAM.

PorthoMCL identified 763,506,331 ortholog gene pairs and identified 230,815 ortholog groups in these genomes. The orthologous pairs (file size: 6.2GB) and orthologous groups (file size: 50 MB) as well as paralogous pairs are available for download at http://ehsun.me/go/porthomcl. We will periodically update our predictions when more genomes are available in the future. The options and arguments needed at each step are discussed in detail in the documentation of the PorthoMCL package that can be freely accessed from github.com/etabari/PorthoMCL.

## Conclusion

PorthoMCL is fast tool with minimal requirements for identifying orthologs and paralogs in any number of genomes. While PorthoMCL uses the same mathematical basis as OrthoMCL to investigate orthology among genomes, it is much faster and a more scalable tool when handling a very large number of genomes than existing tools. PorthoMCL can facilitate comparative genomics analysis through exploiting the exponentially increasing number of sequenced genomes.

## Figures and Tables

**Table 1:** Comparison of runtimes of OrthoMCL and PorthoMCL for different number of genomes

Genomes	Proteins	BLAST Hits	OrthoMCL	PorthoMCL	Speedup
10	19,240	298,647	0:00:18	0:00:11	164 %
	29,912	637,091	0:01:07	0:00:21	319%
	30,111	656,689	0:01:16	0:00:23	330 %
	32,962	721,997	0:01:12	0:00:24	300 %
50	126,020	5,771,483	0:15:55	0:05:55	269 %
	127,724	6,363,917	0:27:53	0:06:08	455 %
	133,974	6,418,035	0:08:29	0:06:15	136 %
	138,258	7,008,798	0:24:06	0:06:18	383 %
100	252,109	18,326,608	1:02:58	0:31:49	198 %
500	1,327,716	283,850,847	-	17:38:55	∞

## Abbreviations

BLAST: Basic local alignment search tool
GB: Gigabytes
HPC: High performance computing
MCL: Markov clustering
MPI: Message passing interface
SQL: Structured query language
